# Urban Pit-Building Insects Are Attracted to Walls for Multiple Reasons

**DOI:** 10.3390/biology10070635

**Published:** 2021-07-08

**Authors:** Inon Scharf, Tomer Gilad, Yuval Taichman, Aziz Subach

**Affiliations:** School of Zoology, The George S. Wise Faculty of Life Sciences, Tel Aviv University, Tel Aviv 6997801, Israel; tomergilad@mail.tau.ac.il (T.G.); taichman@mail.tau.ac.il (Y.T.); aziz@post.tau.ac.il (A.S.)

**Keywords:** antlion, habitat selection, predator-prey interactions, urban ecology, Vermileonidae, wall ecology

## Abstract

**Simple Summary:**

Wormlions are small fly larvae that dig pit-traps in loose soil to hunt ants and other prey. Their natural habitat is caves, but they are also abundant in Mediterranean cities below man-made shelters, even in thin layers of soil. They are especially common next to building walls. First, we show that wormlions are indeed closer to walls than expected by chance. Next, we tested several explanations for this observation: the possible effect of soil depth, soil particle size, shade, and prey abundance. We could not find a single explanation for the wormlion’s proximity to walls, and in each site, a different set of explanations held true. The final step was to conduct an experiment. We placed wormlions on clear sand either in the center or next to the wall and observed whether they moved after a day. Those placed in the center moved over longer distances, and we interpret this result to indicate that those adjacent to the wall are more satisfied with their location. Our study provides an example for how animals take advantage of human-made changes in the habitat and prosper in urban habitats.

**Abstract:**

Whereas most animals find urban habitats to be inferior to natural habitats, some “urban specialist” species thrive there. Wormlions present such an example. Common in Mediterranean cities, they cluster in thin layers of loose soil below man-made shelters. Wormlions are fly larvae that dig pit-traps in loose soil and hunt small arthropods. Our first aim was to determine whether wormlion pits accumulate next to walls. Wormlion pits were indeed closer to walls than expected by chance at most of the study sites. We examined possible factors behind this apparent preference, combining field observations and experiments, laboratory work, and theoretical analysis. We examined the effect of soil depth, particle size, shade, and prey abundance. Each factor provided a partial explanation for the wormlions’ proximity to walls, but none provided an overall explanation. We developed a spatially explicit simulation model, demonstrating under which conditions wall-adjacent positions are favored. Finally, we created artificial microhabitats, and placed wormlions either in the center or next to the wall. The wormlions in the center moved over longer distances than those next to the wall and did so more in the wall’s direction. The abundance of walls may help to explain the success of wormlions in urban habitats.

## 1. Introduction

Urban environments differ strikingly from their immediate surroundings. On the one hand, cities may feature soil, water, air, light, and noise pollution, as well as human disturbance [1,2,3]. All these factors impact many native species, leading to their disappearance from cities and reducing species diversity there [4,5]. On the other hand, cities moderate inter-seasonal climate fluctuations and provide food and water year-round, functioning as “pseudo-tropical bubbles” [6]. This greatly increases the abundance of specific species, which are similar across cities but differ from those in the natural surroundings [7,8]. These “urban” species are often opportunistic in their diet and are pre-adapted to life in cities [9,10]. Such pre-adaptations include high cognitive and innovation abilities, an ability to ascend steep structures such as buildings, and large litter sizes [11,12,13,14]. Such species are also able to exploit the artificial habitat of a city if it resembles their habitat in the wild [15]. Cliff-nesting birds such as swifts and kestrels, which nest in buildings, serve as an excellent example [16,17].

Urban habitats are characterized by a high density of man-made buildings. Such buildings serve as habitats for hundreds of arthropod species, commensal with humans indoors [18]. Another common wildlife habitat in cities is that of the area on or adjacent to walls [19], and some animals settle on building walls or next to them. For example, some spiders prefer constructing their webs on walls, especially in cracks and cavities, which provide protection from wind and rain [20,21]. Walls are often artificially lit and spiders prefer lit habitats because their insect prey are attracted to light [22,23]. Walls also provide suitable habitats for some plant species, depending on the available substrate, minerals, and humidity; and walls are especially suitable for plants naturally occurring next to cliffs or rocks [24]. Small animals capable of climbing are also common on walls, such as the common house gecko [25].

Trap-building arthropod predators select an ambush site and construct a trap to hunt their prey [26,27]. The trap slows down fast-moving prey, increases the predator’s attack range, and enables the predator to hunt relatively large prey [28]. Because constructing a trap is costly [29,30] and relocation is expensive [31], such predators should carefully choose a suitable ambush site. These sites should answer species-specific microclimatic requirements, enable the construction and easy maintenance of efficient traps, and be sufficiently rich in prey [32,33,34]. Trap-building predators and web-building spiders in particular are common in cities, where they construct larger traps and reach larger sizes [35,36,37]. The reason may be the similarity of urban habitats to the natural, open habitat of such predators or the attraction of prey at night by artificial light. Wormlions (Diptera: Vermileonidae) are fly larvae that dig pit-traps in loose soil to ambush arthropod prey. Their hunting method is convergently similar to that of pit-building antlions (Neuroptera: Myrmeleontidae) [38,39]. Wormlions are abundant in Mediterranean cities, in higher densities than antlions [38,40]. They prefer fine, dry, deep, and shaded soil over coarse, wet, shallow, and lit soil when choosing where to dig a pit [41,42,43]. Cities provide large areas of shaded, open ground with a layer of soil and sparse vegetation, suitable for pit construction. In addition, there are more small ants in urban sites of wormlions compared to natural wormlion sites [44], providing hunting opportunities for wormlions, which hunt smaller prey than antlions of a similar size [39]. Similar to some web-building spiders, wormlions in the city construct larger pit-traps and reach larger sizes compared to populations in more natural habitats [44].

A recent study has suggested that wormlions prefer to dig pits next to walls or other barriers, but no quantitative support was provided for this suggestion regarding the wormlion’s natural habitat [45]. In a series of lab experiments, wormlions chose to dig pits next to walls if these provided some shelter from direct light [45]. There are four possible reasons why pit positions next to walls should be preferred: (a) walls may provide shade for at least part of the day; (b) loose soil may accumulate next to walls driven by the wind and function as a “sand fence” [46,47], resulting in deeper soil in which to dig; (c) because wind carries fine particles more easily than coarse ones [48], sand next to walls may be finer than in other locations. Wormlions prefer finer sand [41,49]; and (d) prey may be more abundant next to walls, either because the wall-adjacent microhabitat provides better conditions, or because the prey that reaches the wall moves along it, presenting more hunting opportunities for wormlions. Many insects move along barriers when encountered, demonstrating “wall-following behavior” [50,51,52,53].

Our first aim was to determine whether wormlions in the city are indeed more common next to walls than expected by chance. Next, we examined a series of potential explanations for the detected proximity of wormlions to walls: whether, next to walls (a) the soil is deeper; (b) the soil is finer; (c) it is more shaded; (d) wormlions dig larger pits; and (e) there is a higher abundance of potential prey. Additionally, we designed an individual-based simulation model that simulates the prey movement and offers a behavioral mechanism for why pit-traps next to walls capture more prey. Finally, we conducted a field experiment, examining the tendency of wormlions to relocate when initially placed next to a wall vs. distant from a wall.

## 2. Materials and Methods

### 2.1. Study Sites and Wormlion Zones

**Figure 1:**
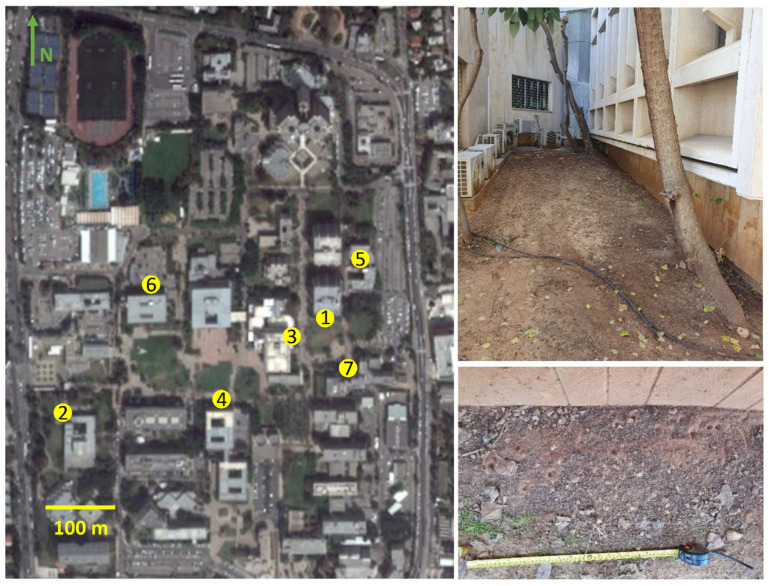
Clockwise: A photo of Tel Aviv University (Tel Aviv, Israel, from Google Earth) with the seven study sites marked. An overview of one site (Schreiber building), and the wormlion pits adjacent to the building’s wall. The numbers stand for sites: (1) Britannia, (2) Buchmann-Mehta, (3) Central library, (4) Gilman, (5) Green, (6) Mexico, and (7) Schreiber.

Wormlions are abundant in urban habitats in loose soil below man-made covers. Wormlions reach higher densities than the ecologically similar pit-building antlions [38], possibly owing to much lower rates of cannibalism. That said, competition over suitable sites is evident, with large wormlions being superior over small ones [54]. We chose seven sites at Tel Aviv University, Tel Aviv, Israel (Figure 1; Figure S1 in the Supplementary Material), which we refer to according to the names of the adjacent university buildings: Britannia, Buchmann-Mehta, Central library, Gilman, Green, Mexico, and Schreiber. The distance between the two closest and the most distant sites is 60 m and 470 m, respectively. The wormlion zones captured in each photo occupy from 55 × 35 cm to 110 × 110 cm in area. At each site, we took 1–3 photos, counted the number of pits, and measured the distance of each pit to the nearest wall (using ImageJ [55]). The sites differed in various aspects, such as the geometry of the surrounding buildings, soil depth, illumination level, orientation, and wormlion density. We therefore statistically examined each site separately. To determine whether the wormlion pits were closer to the walls than expected by chance, we conducted a bootstrap procedure in MATLAB (v. 2019). We created 1000 groups of distances (same in size) of the observed distances, allowing each distance to be chosen more than once. We calculated for each group the mean distance from the wall. If the observed mean was below or above the 95% confidence intervals of the means of the simulated groups, the pits were either closer to or farther from the wall than expected. If not noted otherwise, statistics were conducted using SYSTAT 13 (Systat Software, San Jose, CA, USA).

### 2.2. The Link Between Soil Depth, Distance from the Wall, and Pit Area

To determine whether there is a link between soil depth and pit distance from the wall, we measured the soil depth using a digital caliper (accuracy of 0.01 mm). We chose the center of each photo at each site, drew two transects vertically to the wall with a distance of 10 cm in between, and measured soil depth at 9–11 points in each transect (18 to 22 points in total). We used separate one-way linear regressions for each photo to analyze these data. We used a Cohen’s Kappa test to determine whether the probability of wormlions being closer to the wall (scored as 1 or 0) is linked to a negative slope of soil depth (scored as 1 or 0). Next, we chose four sites (Britannia, Green, Mexico, and Schreiber; Figure 1), and selected 25 wormlion pits at varying distances from the wall. We photographed each pit and measured its area and soil depth, collected the wormlion, weighed it in the lab (Precisa, Dietikon, Switzerland; accuracy of 0.1 mg), placed it in fine, deep sand (particle size < 250 µm, depth of 5 cm), allowed it to construct a pit, and photographed and measured the pit area after 24 h. We conducted the following analysis, separately per site: (1) linear regressions on wormlion mass vs. the distance from the wall, to determine whether the larger wormlions were closer to the wall than expected by chance; (2) linear regressions to determine the effect of the distance from the wall, soil depth, and wormlion body mass on the pit area in the field; and (3) examination of the effect of the same factors on pits constructed in the lab. The reason for measuring pits in the lab was to determine whether those factors affecting the pit area in the field also did so in the lab. Pit areas and body mass were square-root transformed because they deviated from a normal distribution. See the Supplementary Material Figure S2, for correlations of the areas of pits constructed in the field and the lab.

### 2.3. Measurements of the Soil Particle Sizes

We collected sixteen samples of 150 mL soil from four of the seven sites (Green, Gilman, Mexico, and Schreiber); eight next to the wall and eight 40 cm from the wall. The soil samples were dried for 48 h at 60 °C and then sieved through five sieves (Ari Levy, Petach Tikva, Israel) allowing only particles in descending size to pass (710, 500, 250, 105, and 63 µm; similar to [41,56]). We then weighed the soil of the six obtained particle size ranges. Two examinations are of particular interest. First, the majority (~85%) of soil particles at wormlion sites were smaller than 540 µm [41]. We therefore divided the mass of particles larger than 500 µm by the total sample mass (hereafter, “proportion of large particles”). Second, the two most common particle sizes were 250–500 and 106–250 µm. We therefore divided the mass of particles smaller than 250 µm by the total sample mass (hereafter, “proportion of small particles”). We used two one-way ANOVAs per site (on arcsin-transformed values), with positions as the explanatory variable and either one of the two proportions as the dependent variable, to examine for differences.

### 2.4. Light Measurements

On two sunny days, in early March and late May, we used a light-meter (Digi-Sense 20250−00, Dakota Instruments, Orangeburg, NY, USA) to measure light intensity to determine whether positions adjacent to the wall were more shaded. We measured light at five points adjacent to the wall and five points at a distance of 40 cm from the wall at each of the seven studied sites. We repeated this procedure three times, at 08:30 (morning), 12:30 (noon), and 16:30 (afternoon). We analyzed the data, separately per day and site, using a two-way ANOVA, with the time, position (adjacent to the wall or 40 cm away from the wall), and the interaction as explanatory variables, and light intensity as the response variable. Light intensity was log-transformed due to its deviation from a normal distribution.

### 2.5. Arthropod Abundance

To determine whether prey are more abundant next to walls, we focused on two sites, Green and Schreiber, and placed a total of 240 artificial pitfalls (diameter of 5.5 cm), spread over two successive days in April, and at two sites, of which 120 pitfalls were adjacent to the wall and 120 were at a distance of 40 cm from the wall (Supplementary Material Figure S3). Each pitfall trap was open for 24 h, after which we collected all arthropods (and released any other animals). We counted all individuals and sorted them into arthropod orders. Most of the captured arthropods were ants, which we then sorted to the genus/species level. Assuming that ants are the main prey of wormlions [57], we used χ^2^ tests to determine whether there is a difference between microhabitats next to the wall and those 40 cm away from the wall in the overall number of arthropods captured.

### 2.6. Simulation Model: Prey Movement Affects Capture Success

We designed a spatially explicit individual-based model (in Matlab v. 2019) to examine the capture probability of ambush predators located next to the wall vs. other positions. See the Supplementary Material Figure S4, for a flowchart. We used a square grid of 50 × 50 cells, with one side referred to as a “wall”. We placed four ambush predators in the grid, two in random positions and the other two next to the wall (in random positions along it). The predators never moved. Fifty prey individuals were then placed in random positions on one of the three non-wall sides and moved each time step in one of four directions (forward, backward, right, and left). The initial movement direction was randomly determined, but each prey individual then had a 60% probability of continuing to move in the same direction, and a 20% probability of turning to either side (a turn of 90°). When the prey reached a grid cell occupied by a predator, it was captured, and a new prey appeared on the next time step, to retain a fixed number of prey. When a prey individual left the grid, it reappeared on one of the three non-wall sides, in random positions (“absorbing boundaries”; [58]). Assuming that the prey demonstrated a wall-following behavior (see Introduction), a prey that reached the wall kept moving along the wall until it left the grid. The number of prey caught by each predator category (adjacent to the wall vs. random position) was averaged. The simulation was run 30 times, with each run lasting 200-time steps, after which we used a bootstrap procedure on the Δsuccess of the two predator types (number of prey caught by the predators ambushing next to the wall minus those caught by predators ambushing at random positions) to determine the 95% confidence intervals (similar to [58]). To further explore the conditions under which predators adjacent to the wall may be more successful than those in random positions, we modified several of the simulation parameters in a full-factorial design (3 × 3 × 2 treatment combinations): (1) two, six, or ten predators, half of which ambush next to the wall and from random positions; (2) 50, 100, or 150 prey items; and (3) the prey has a probability of either 60% or 90% of maintaining its current movement direction, resulting in either less or more directional movement. Next, we modified the ratio of predators next to the wall, which until now had been fixed at 50%. We used either 6 or 10 predators in total, two directionality levels of the prey (60% or 90%, see above), and either 30–33% of the predators next to the wall (2 and 3 for 6 and 10 predators in total, respectively) or 67–70% predators next to the wall (4 and 7 for 6 and 10 predators in total, respectively), summing-up to eight treatment combinations.

### 2.7. Experiment: Wormlion Relocation as a Function of Distance from the Wall

We placed 1.5 cm deep soil on the concrete ground below a roof (~90 × 50 cm in area), in a place potentially suitable for wormlions, but from which they had been previously absent due to the lack of loose soil. This procedure was repeated at four times in four mini-sites, next to the Britannia site. For each replication, we collected 40 wormlions from nearby, 20 of which we placed adjacent to the wall, and the other 20, 40 cm away from the wall (a total of 160 wormlions). The distance between adjacent individuals was 5 cm. We let the wormlions construct pits for 24 h, after which we photographed the site, including the constructed pits. We measured for each wormlion in each treatment the distance from the initial transect position (either the wall or a transect 40 cm away from the wall). To determine whether individuals located next to the wall or distant from the wall moved over longer distances, we used a mixed linear model, with distance from the initial position as the response variable, treatment (initial position) as the fixed explanatory variable, and replication as a random variable. The distance was log_10_-transformed, as it deviated from a normal distribution. In order to determine whether wormlions showed a trend to move towards a wall, we examined whether wormlions placed in the center moved more frequently towards or away from the wall after 24 h, using a χ^2^ test.

## 3. Results

### 3.1. Study Sites and Wormlion Zones

**Figure 2:**
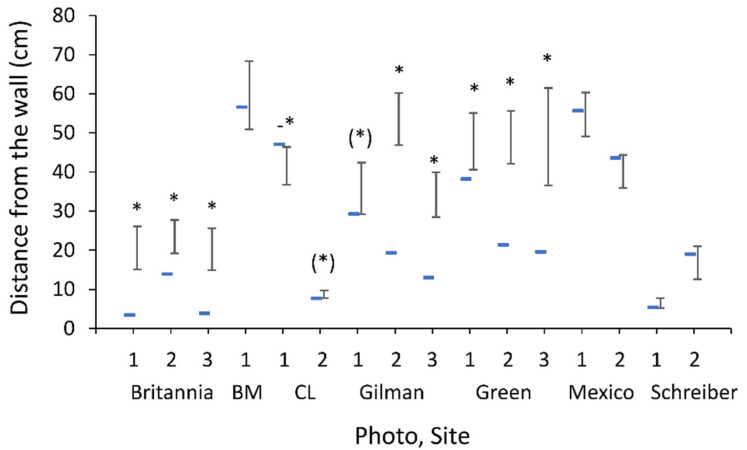
The observed mean distance from the wall (horizontal line) and the expected distance (vertical lines, 95% confidence intervals, determined by bootstrap), separately per site (name) and photo (number). BM and CL stand for Buchmann-Mehta and Central Library. *, (*), and -* stand for an observed distance shorter than expected by chance, a marginally non-significant result in the same direction, and an observed distance longer than expected by chance.

Table 1 presents a summary of the area, number of wormlions, density, the mean distance from the nearest wall, and whether pits were closer to the wall than expected by chance, per photo per site. At three of seven sites, there was at least one photo documenting wormlions closer to the wall than expected. Wormlions were closer to the wall than expected in eight out of 16 photos, with an additional two photos demonstrating a non-significant trend (Figure 2). In one photo, wormlions were more distant from the wall than expected.

### 3.2. The Link between Soil Depth, Distance from the Wall, and Pit Area

The soil was deeper next to the wall in five of 16 photos (Table 1). While this is a relatively high proportion, there was no link to the probability of wormlions being closer to the wall than expected (k = 0.059, *p* = 0.790). There was also no link between the distance from the wall and the wormlion body mass for three of the four sites examined (Green, Mexico, and Schreiber; *p* > 0.240 for all three), while for one site wormlions next to the walls were larger than those at a distance (Britannia; t = −2.548, *p* = 0.018). Body mass was positively correlated with pit area in both the field and lab for all sites tested (all sites; *p* < 0.046). Soil depth had no effect on pit area (all sites; *p* > 0.253). In contrast to our expectation, distance from the wall was positively (and not negatively) correlated with pit area, but only at one site (Mexico; t = 3.496, *p* = 0.002). See Table S1 in the Supplementary Material for the full statistics.

### 3.3. Measurements of Soil Particle Sizes

**Figure 3:**
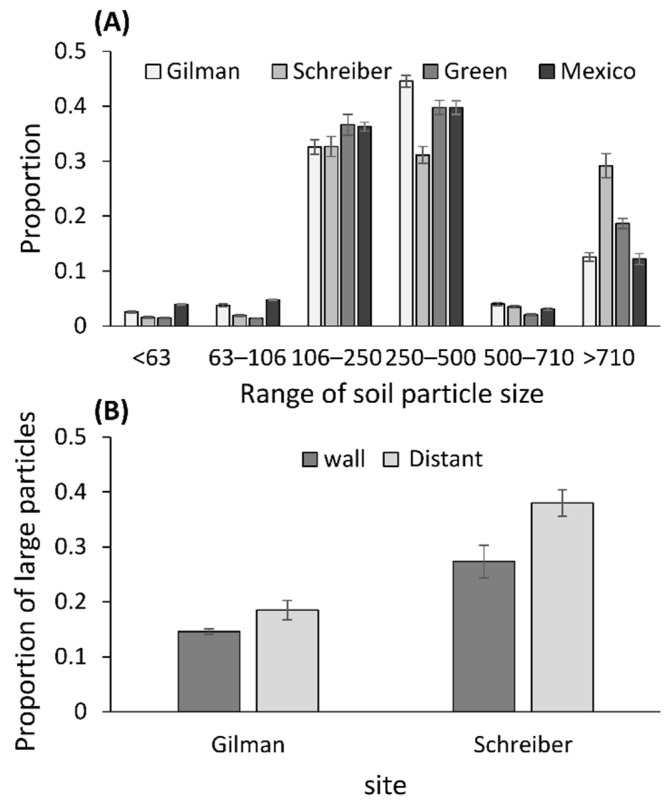
(**A**) The range of soil particles at four sites. (**B**) At two of the four study sites, the proportion of large particles at distant positions was higher than that next to walls. Means ± 1 SE are presented.

The proportion of large particles was higher next to the wall at two of the four sites examined (Figure 3; Gilman: F_1,14_ = 4.620, *p* = 0.0496; Schreiber: F_1,14_ = 8.025, *p* = 0.013; Green: F_1,14_ = 0.401, *p* = 0.537; Mexico: F_1,14_ = 0.166, *p* = 0.690). At only one of these two sites (Gilman) were wormlions closer to the wall than expected. The proportion of small particles was similar across positions in all four sites (Gilman: F_1,14_ = 1.769, *p* = 0.205; Schreiber: F_1,14_ = 2.719, *p* = 0.121; Green: F_1,14_ = 0.364, *p* = 0.556; Mexico: F_1,14_ = 0.034, *p* = 0.857).

### 3.4. Light Measurements

**Figure 4:**
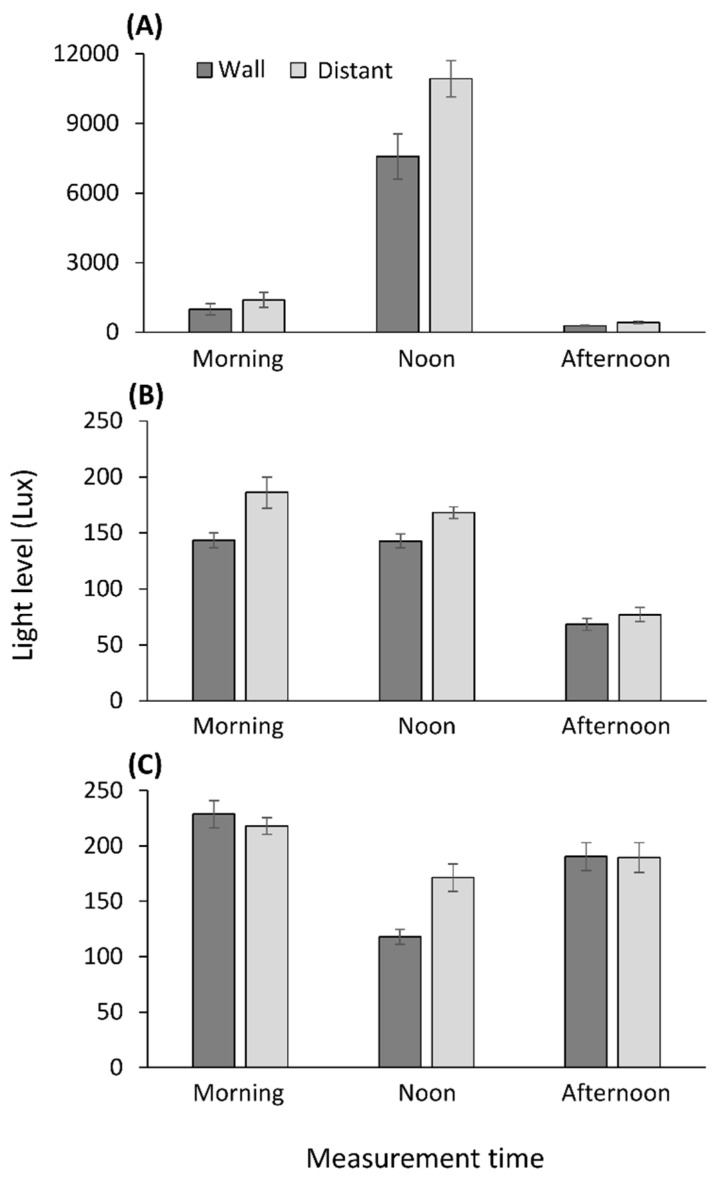
Light levels at three of the studied sites ((**A**) Britannia, (**B**) Schreiber, (**C**) Green), next to the wall and at more distant positions. While the wall positions were usually more shaded, at the Green site position interacted with daytime to affect the light level. Means ± 1 SE are presented.

Light intensity changed with time at all sites (March: Table 2; May: Supplementary Material Table S2). In both March and May, the same four of the seven sites, positions next to the wall were more shaded than at 40 cm distance from the wall (either throughout the day or partially), whereas at one site the opposite held true. See Figure 4 for three sites in March. At two of the four sites, where the wall provided some shade, wormlions were also closer to the wall than expected. See Table S2 in the Supplementary Material for the results of the second measurement in May, which demonstrated qualitatively identical differences between wall and distant positions. We also demonstrate in Table S2a a general link between surface temperature and light level.

### 3.5. Arthropod Abundance

*Green site:* More arthropods fell into the traps adjacent to the wall than those 40 cm away (χ^2^ = 28.57, df = 1, *p* < 0.001; 229 vs. 128 individuals). The proportion of ants from the arthropods captured next to the wall did not differ according to position (χ^2^ = 2.605, df = 1, *p* = 0.107; 61% vs. 52%). The ants belonged almost solely to two small species: the invasive little fire ant *Wasmannia auropunctata* and the longhorn crazy ant *Paratrechina longicornis* (78% and 19%, respectively, body length < 3 mm). The remaining 3% were also small, and therefore probably hunted by wormlions.

*Schreiber site:* In contrast to the Green site, more arthropods fell into traps distant from the wall than into those next to it (χ^2^ = 46.09, df = 1, *p* < 0.001; 232 vs. 107 individuals). More ants were also captured distant from the wall than next to it (χ^2^ = 61.76, df = 1, *p* < 0.001; 72% vs. 27%, respectively). The captured ants belonged mostly to the small *Pheidole* and *Nylanderia* spp. (80% and 16% of the capture arthropods, respectively), probably also hunted by wormlions. The remaining 4% were mostly *Camponotus*, which are too large for wormlions to handle. See the Supplementary Data for the full list of arthropods.

### 3.6. Simulation Model: Prey Movement Affects Capture Success

**Figure 5:**
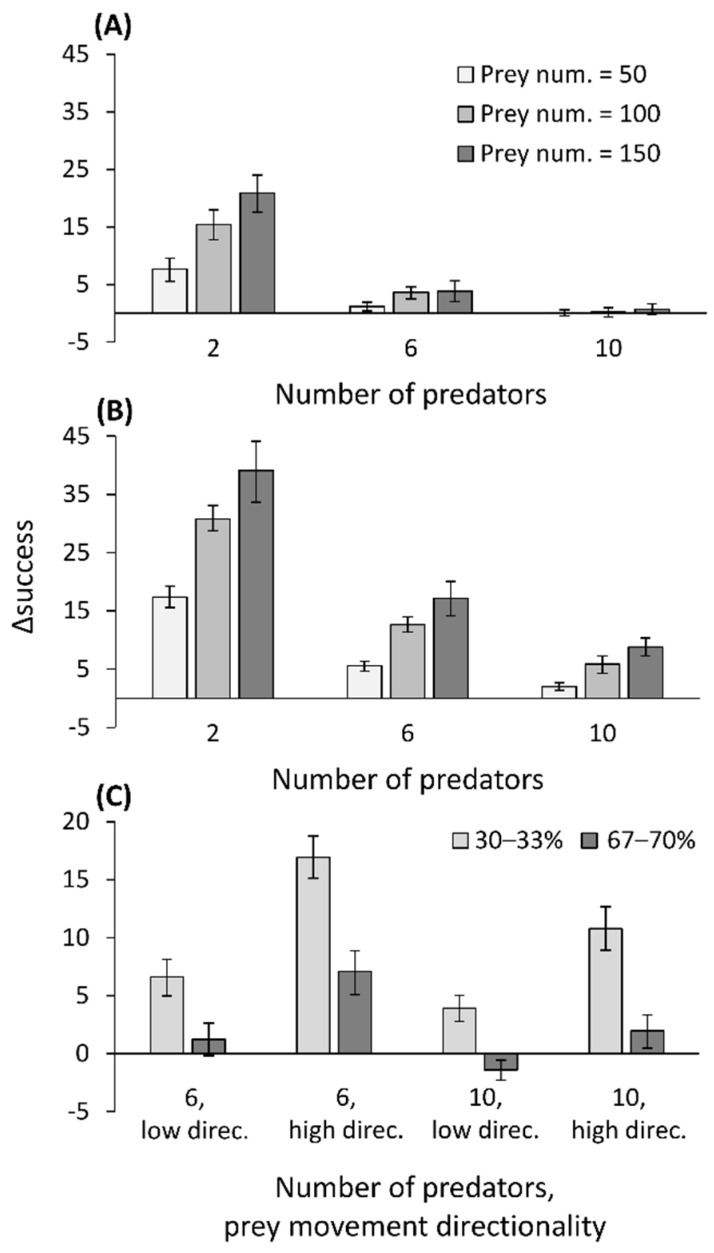
The simulation results. The Δsuccess (the number of prey caught by predators next to the wall minus those caught by predators at random positions) simulating three prey densities (50, 100, and 150 prey), and predator densities (2, 6 and 10), when: (**A**) prey move less directionally (a probability of 0.6 to keep moving in the initial direction), or (**B**) more directionally (a probability of 0.9 to keep moving in the initial direction). (**C**) A change of the ratio of predators next to the wall vs. predators in random positions (ca. one-third vs. ca. two-thirds), using two predator densities (6 and 10) and low and high directionality of prey movement (low direc. and high direc. stand for low and high movement directionality of the prey). Means and 95% confidence intervals are presented.

The simulation highlights the scenarios under which ambushing prey next to the wall is more successful. In brief, the superiority of a wall position increased with an increase in the number of prey, a decrease in the number of predators, an increase in the directionality level of the prey (Figure 5a,b), and a decrease in the ratio between the predators next to the wall and the total number of predators (Figure 5c). However, certain scenarios could even end in the inferiority of wall positions (e.g., 10 predators, low directionality level of prey, high ratio of wall positions).

### 3.7. Experiment: Wormlion Relocation as a Function of Distance from the Wall

**Figure 6:**
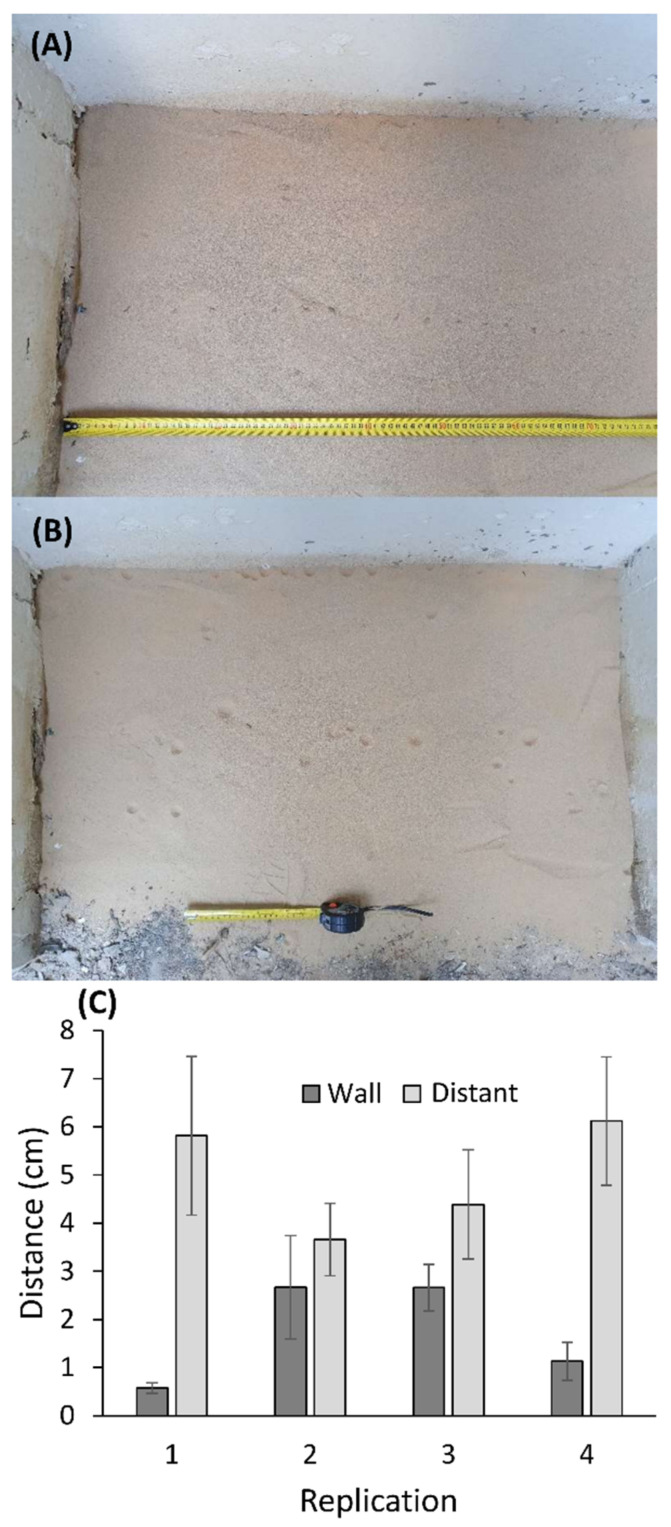
The experiment examining wormlion relocation as a function of the distance from the wall, (**A**) immediately after placing the wormlion, and (**B**) 24 h later. (**C**) The distance moved depending on the initial position, presented separately per replicate. Means ± 1 SE are presented.

Wormlions that were placed next to the wall moved over shorter distances (~35%) than those placed at a distance from the wall (t = 5.233, *p* < 0.001; Figure 6). When relocating, wormlions placed away from the wall moved more frequently in the wall’s direction than away from it (45 of 73 pits were closer to the wall than the initial placement location, while the rest were more distant: χ^2^ = 3.959, df = 1, *p* = 0.047).

## 4. Discussion

We have demonstrated here that wormlions construct their pit-traps next to walls more than expected by chance, although this tendency is site-dependent and does not involve the construction of larger pits. We examined several possible reasons for this apparent preference. Most of the studied factors presented a partial explanation. At some sites, the soil was deeper next to walls than it was further away, but this pattern was not correlated with the wormlion’s apparent preference for walls. Similarly, positions next to walls were more shaded at some sites, but here too there was not a clear correlation with the apparent preference for walls. In two of four examined sites, the soil next to the walls was finer than soil further away, but only at one such site were the wormlions closer to the wall than expected by chance. There may be a correlation between the wormlion preference for wall-adjacent positions and the abundance of prey there, but such a pattern is also site-dependent. The simulation provided a mechanism explaining why arthropod availability may be higher next to walls and highlighted the conditions under which a wall should be preferred. Finally, wormlions placed next to walls relocated less frequently than those placed distant from walls, suggesting that wormlions perceive wall positions as superior over less adjacent positions. Consequently, there appears to be several not mutually exclusive explanations for the apparent preference for wall-adjacent positions.

Wormlions were closer to walls than expected by chance at three of the seven sites examined (eight of the 16 photos, plus two photos exhibiting a trend). Wormlions, whose natural habitat is mostly caves, take advantage of man-made covers providing shelter from direct sunlight and rain [44]. This adds to a growing list of species which successfully use urban habitats, depending on either their ability to adapt to such habitats, existing pre-adaptations, or phenotypic plasticity [59]. It seems like trap-building predators fit well in urban habitats, either because the city offers them suitable habitats to which they are pre-adapted or owing to their opportunistic foraging and greater hunting opportunities in cities (see Introduction). Wormlions are an additional example of a successful trap-building predator in cities. Wormlions are not the only species using walls or wall-adjacent microhabitats in cities, and other examples include a variety of plant, bird, lizard, and arthropod species [17,19,21,60]. Walls, in contrast, provide worse conditions for amphibians in ponds compared to ponds with no vertical walls, so the advantage walls provide is species-specific [61].

In six of the 16 photos, inconsistently within sites, the soil was deeper next to the wall, possibly due to the walls functioning as “sand traps”. Walls with deeper soil, however, did not attract more wormlions. In lab experiments, wormlions preferred 1–2 cm-deep sand over 0.5 cm-deep sand [42,62], but pits constructed in sand deeper than 1.5 cm did not differ in size from those constructed in deeper sand (up to 3 cm; [56]). This suggests that the deeper soil next to walls was relevant in only three of the five photos because the minimal soil depth in the two other photos was above 3 cm. We therefore conclude that soil depth is only a partial explanation for wormlion attraction to walls. Wormlions also prefer fine sand over coarse sand [63,64]. If the wind is responsible for the presence of deeper soil next to walls, the soil there should be finer, providing more suitable sites for wormlions. At two of the four examined sites, finer soil was indeed found next to the walls, partially supporting such an explanation. However, only at one of these sites were wormlions closer to the wall than expected. Soil-dwelling insects and Dipterans, in particular, can be used as bioindicators for soil quality [65,66]. The study of the soil traits that facilitate the abundance and richness of such insects is therefore of interest. For example, species richness of soil-dwelling insects increases with the content of organic matter, and first increases but then decreases with humidity [67].

Shade could present a better explanation for the wormlion apparent preference for walls. First, a series of lab experiments has demonstrated that wormlions prefer walls when these provide shade [45]. Second, wall positions were more shaded, at least temporarily, than positions more distant from walls at four of the seven sites examined. Many other sit-and-wait predators in warm regions, such as snakes hunting lizards or rodents, prefer to ambush prey under shade, either in order not to get overheated, or because prey are more abundant under shade, seeking shelter as well [68,69]. However, at only two of these sites were wormlions closer to the wall than expected, making this explanation partial. Furthermore, while wormlions prefer shaded sand over lit sand in the lab, the ability of wormlions to sense favorable conditions diminishes even two cm away [70]. It is also unknown how sensitive wormlions are to slight differences in illumination, smaller than the ones previously examined in the lab [42].

Regarding soil particle size, at two of the four sites examined, the soil next to the wall was finer, while no pattern was detected at the other two sites. Only at one of the two former sites were wormlions closer to the wall than expected. We conclude that while finer soil may accumulate better next to walls, it is not the cause of wormlion attraction. Wormlions prefer fine sand, with smaller wormlions being much choosier than larger ones, but both are limited in their perceptual range [56,62,63]. It is not surprising that other soil-dwelling insects demonstrate some preference for specific size ranges of soil particles too. For example, a beetle and a fly species prefer coarse-grain soil for pupation or oviposition [71,72]. A future experiment should examine the effect of wind not only on the accumulation of sand, but also directly on wormlions, as a source of disturbance to pit construction and maintenance. If walls protect against wind, requiring less frequent pit maintenance, it may be another reason for the suggested preference for walls.

Arthropod abundance may be a more convincing explanation. First, at a site in which wormlions were closer to the wall than expected, arthropods were more abundant next to the wall. In contrast, at a site in which wormlions showed no preference for the wall, arthropods were not more abundant next to it. Most of the arthropods captured were small ants, presenting a suitable prey for wormlions, which hunt smaller prey than do antlions [39]. The simulation model offers a mechanistic explanation for how more ants may fall into pits adjacent to walls. First, the advantage that wall positions provided in capture success increased with prey abundance because it stretched the existing differences between the two positions. Second, wall positions were superior when prey moved directionally and had a higher probability of reaching the wall before exiting the arena from its sides. When prey reached the wall, they kept moving next to it, increasing their encounter rate with predators. Third, with increasing predator density, or the ratio of predators ambushing prey next to walls out of their total number, the advantage of wall positions declined, leading to a frequency-dependent success of predators next to walls. The reason for this is that of the interception of prey by predators next to the wall that was also closer to the arena’s periphery (“shadow competition”; [73]). This is important, because it demonstrates that positions next to the wall, which are usually superior to random positions in space, may turn over and become inferior, depending on their frequency. In other words, the decision of which strategy to employ should depend on that of nearby conspecifics. The simulation assumes that insects reaching the wall follow it, as has been shown in ants and other insects (see Introduction). This simulation is quite general and can fit any sit-and-wait predator ambushing moving prey. An example for such cases is the timber rattlesnake ambushing prey next to fallen logs [74]. It is profitable to ambush there, because such logs serve as runways for small rodents, which move along them [75].

The experiment of placing wormlions next to the wall vs. at a distance from it demonstrated that wormlions relocated over larger distances when placed away from the wall. This result builds on previous studies demonstrating active microhabitat selection by pit-building predators (e.g., [42,76]). It suggests that the apparent preference for walls is achieved by the larvae themselves. It remains to be tested whether ovipositing females lay more eggs next to walls, demonstrating a match between the adult female and larval habitat selection like other insects (e.g., antlions and dragonflies/damselflies [77,78]). Wormlions more distant from the wall relocated more until they reached a wall. Although there is no need to sense the wall from a distance in order to approach it, the experiment revealed a tendency for wormlions placed at a distance from the wall to move towards it. It is unknown how wormlions sense the wall, especially since lab experiments demonstrate that the wormlion ability to sense the shade, as well as other favorable microhabitat traits (e.g., deep and dry sand) deteriorate fast even at a distance of a few cm [62,70]. Nevertheless, those lab experiments did not apply a gradient of illumination, but simply split the test arena into two halves. In nature, there may be a gradient of light, from more lit central positions towards the more shaded wall. If this holds true, wormlions may follow this gradient and gradually approach positions next to walls. In short, it remains to be uncovered if and how exactly wormlions perceive the wall from a distance.

## 5. Conclusions

Wormlions are often located closer to walls than expected by chance. They relocate more frequently when placed away from a wall than when next to a wall and move in slightly higher proportions towards the wall than away from it. Although we carefully sought a single, overall explanation for why wormlions prefer positions next to walls, we did not find one. Rather, a combination of explanations was found, differing in dominance among sites. The two most convincing explanations are those of shade next to walls, and a higher abundance of potential prey next to walls. The presented simulation suggests a mechanism related to prey movement along the wall, potentially explaining why wormlions closer to the wall capture more prey. It will be fruitful to further examine the simulation prediction and to determine whether wormlions next to walls indeed capture more prey. The excess of walls is probably one of the reasons why wormlions excel in urban habitats.

## Figures and Tables

**Table 1:** The site names, photo numbers, orientation, wormlion zone area, number of pits, soil depth (mean ± 1 SD, range), and the slope (and *p* value) of soil depth vs. the distance from the wall, per photo per site.

Site, Photo, Orientation	Area (cm), Pit #	Soil Depth (mm), [Range]	Slope, *p* Value
Britannia, 1, S	65 × 50, 24	9.3 ± 4.2, [3.3, 17.8]	−0.070, 0.069
Britannia, 2, S	95 × 60, 49	12.4 ± 4.7, [6.2, 27.2]	−0.066, 0.077
Britannia, 3, S	70 × 55, 25	7.5 ± 6.4, [2.9, 22.1]	−0.137, **<0.001**
Buchmann-Mehta, 1, W	120 × 90, 61	51.2 ± 29.6, [12.9, 114.5]	0.655, **<0.001**
Cent. library, 1, N	85 × 75, 86	36.8 ± 13.6, [18.3, 72.7]	−0.111, 0.239
Cent. library, 2, E	60 × 45, 90	90.6 ± 42.8, [22.0, 150.0]	−1.189, **0.011**
Gilman, 1, N	105 × 75, 41	54.4 ± 31.5, [10.6, 144.8]	0.156, 0.575
Gilman, 2, N	105 × 75, 85	57.6 ± 18.9, [31.8, 100.8]	−0.477, **0.001**
Gilman, 3, N	105 × 70, 44	82.1 ± 35.1, [28.4, 150.0]	−0.078, 0.827
Green, 1, S	100 × 100, 50	31.1 ± 20.3, [8.2, 84.8]	−0.350, **0.008**
Green, 2, S	100 × 100, 59	35.0 ± 26.2, [12.8, 105.8]	−0.095, 0.606
Green, 3, S	100 × 100, 21	38.3 ± 36.9, [12.4, 150.0]	0.183, 0.479
Mexico, 1, N	110 × 110, 112	33.2 ± 15.9, [10.3, 70.8]	−0.359, **<0.001**
Mexico, 2, N	110 × 80, 121	37.1 ± 14.8, [7.9, 68.7]	−0.126, 0.328
Schreiber, 1, W	75 × 30, 27	75.2 ± 30.7, [28.5, 140.0]	0.019, 0.974
Schreiber, 2, E	55 × 35, 21	127.5 ± 32.2, [53.8, 150.0]	0.491, 0.420

Significant slopes (soil depth vs. distance from the wall) are in bold. If the slope is significant, the soil more distant from the wall is either shallower or deeper (negative or positive slope, respectively).

**Table 2:** Statistics for the change in illumination with daytime in early March (M = Morning, N = Noon, A = Afternoon) and the difference between positions next to the walls and distant from the walls (W, D). At each site, the three rows stand for each explanatory variable tested: time (df = 2, 24), position (1, 24), and their interaction (2, 24). The order of letters indicates how daytimes and positions differ (larger values first). Significant results appear in bold.

Site	F	*p*
Britannia	246.867 9.788 0.043	<**0.001** N, M, A **0.005** D, W 0.958
Buchmann- Mehta	3.887 8.065 1.246	**0.035** N, A, M **0.009** W, D 0.306
Central library	10.921 0.037 0.028	<**0.001** N, A, M 0.848 0.972
Gilman	91.806 0.037 0.507	<**0.001** M, N, A 0.849 0.609
Green	27.562 4.475 6.801	<**0.001** M, A, N **0.045** D, W **0.005**
Mexico	76.352 11.599 0.310	<**0.001** M, N, A **0.002** D, W 0.736
Schreiber	92.211 10.456 0.498	<**0.001** M, N, A **0.004** D, W 0.614

## Data Availability

The dataset is attached as an Excel file.

## Supplementary Materials

The following are available online at https://www.mdpi.com/article/10.3390/biology10070635/s1, Figure S1: Photos of all sites studied in the manuscript, Figure S2: A comparison between wormlion pits constructed in the field and the lab, Figure S3: A photo of the pitfall traps to estimate the arthropod abundance, Figure S4: A flowchart of the simulation model, Table S1: Results of the link between the distance from the wall, body mass, soil depth, and pit area in the field and the lab, Table S2: Results of the second measurement of light intensity (late May) and measurements demonstrating a link between illumination and temperature, Excel File S1: The whole dataset.
