# Supplementary material for: Urban Pit-Building Insects Are Attracted to Walls for Multiple Reasons
Source: Biology (Basel). 2021 Jul 8;10(7):635. doi: 10.3390/biology10070635 (PMC8301029; doi:10.3390/biology10070635)
Supplement: Supplementary file 1 [file biology-10-00635-s001.zip › biology-1268196-supplementary.pdf]

Supplementary Material for the manuscript:

Urban pit-building insects are attracted to walls for multiple reasons

Inon Scharf, Tomer Gilad, Yuval Taichman and Aziz Subach

The Supplementary Material comprises five parts:

1. Photos of all sites studied in the manuscript (Fig. S1).
2. A comparison between wormlion pits constructed in the field and the lab (Fig. S2).
3. A photo of the pitfall traps to estimate the arthropod abundance (Fig. S3).
4. A flowchart of the simulation model (Fig. S4).
5. Results of the link between the distance from the wall, body mass, soil depth, and pit area in the field and the lab (Table S1).
6. Results of the second measurement of light intensity (late May; Table S2) and measurements demonstrating a link between illumination and temperature (Table S2a).

1. Photos of all sites included in the manuscript.

The study was based on seven sites at Tel Aviv University, named on the adjacent university buildings. Schreiber site is presented in the manuscript, as an example, and here are the six other ones. For each site, we present an overview photo and a photo of some of the wormlion pits present.

Figure S1a: Britannia.

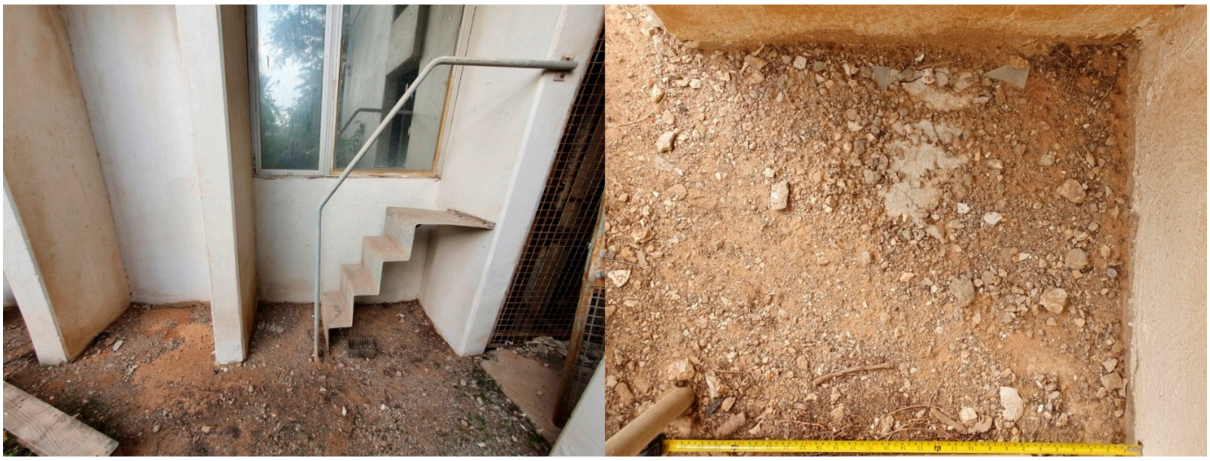

Figure S1b: Buchmann-Mehta.

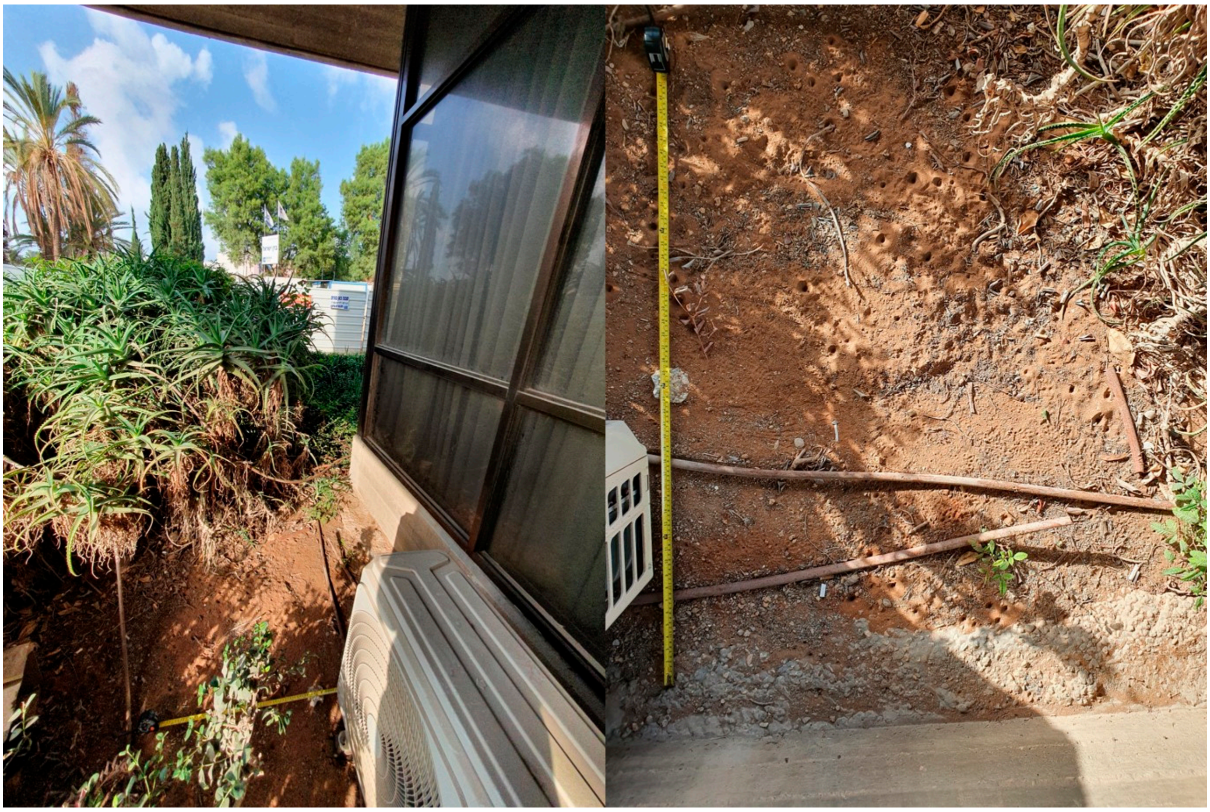

Figure S1c: Central library.

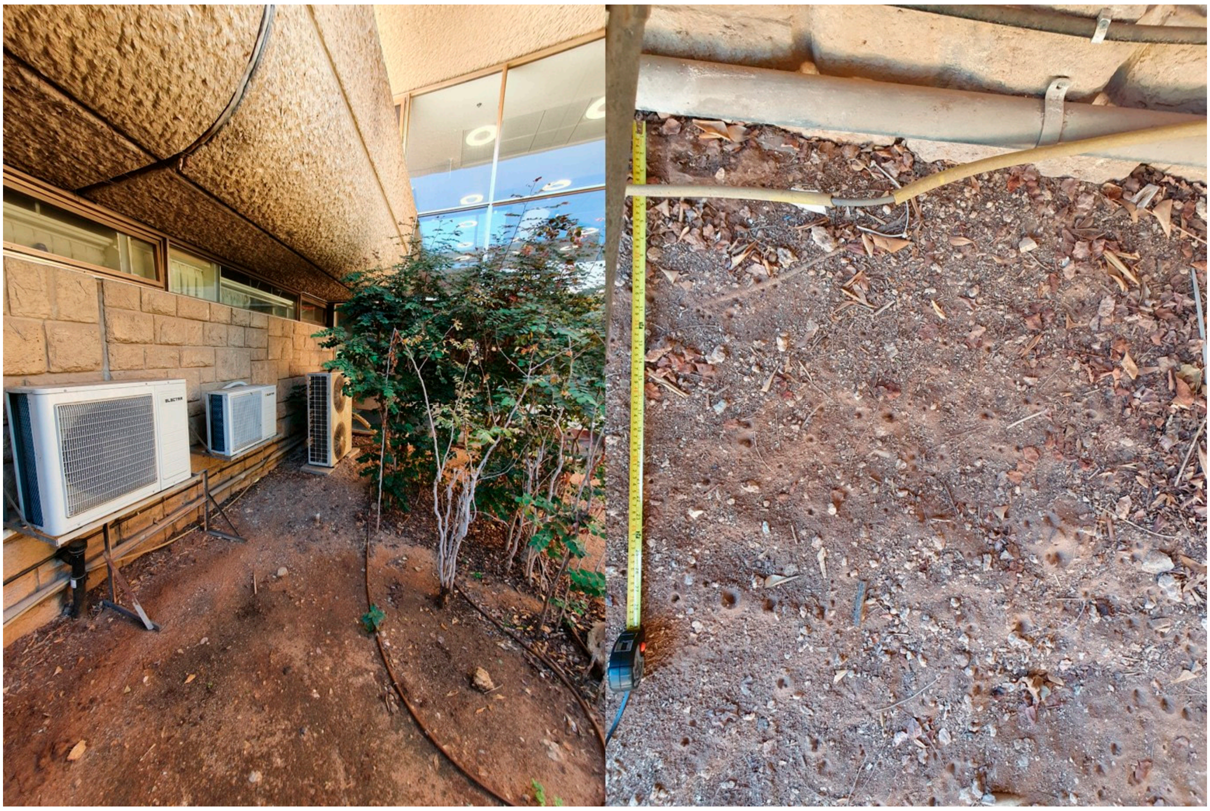

Figure S1d: Gilman.

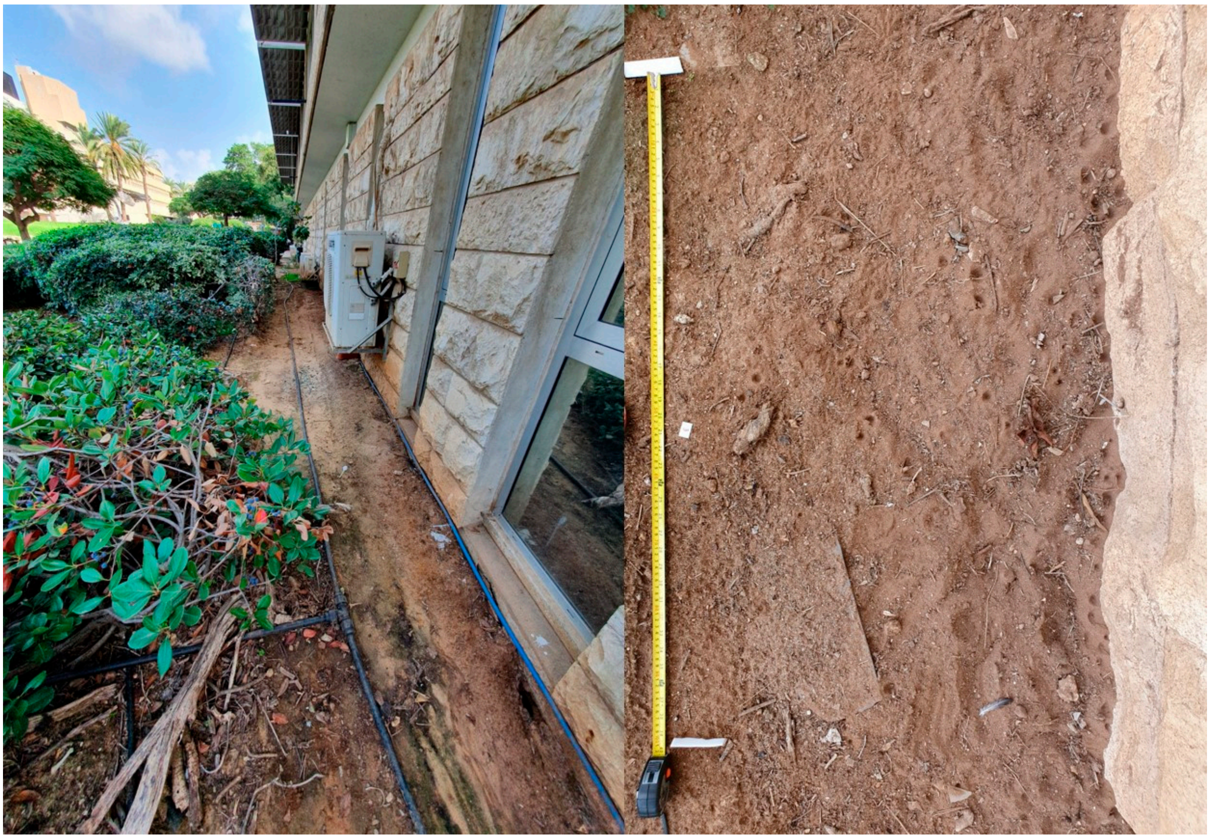

Figure S1e: Green.

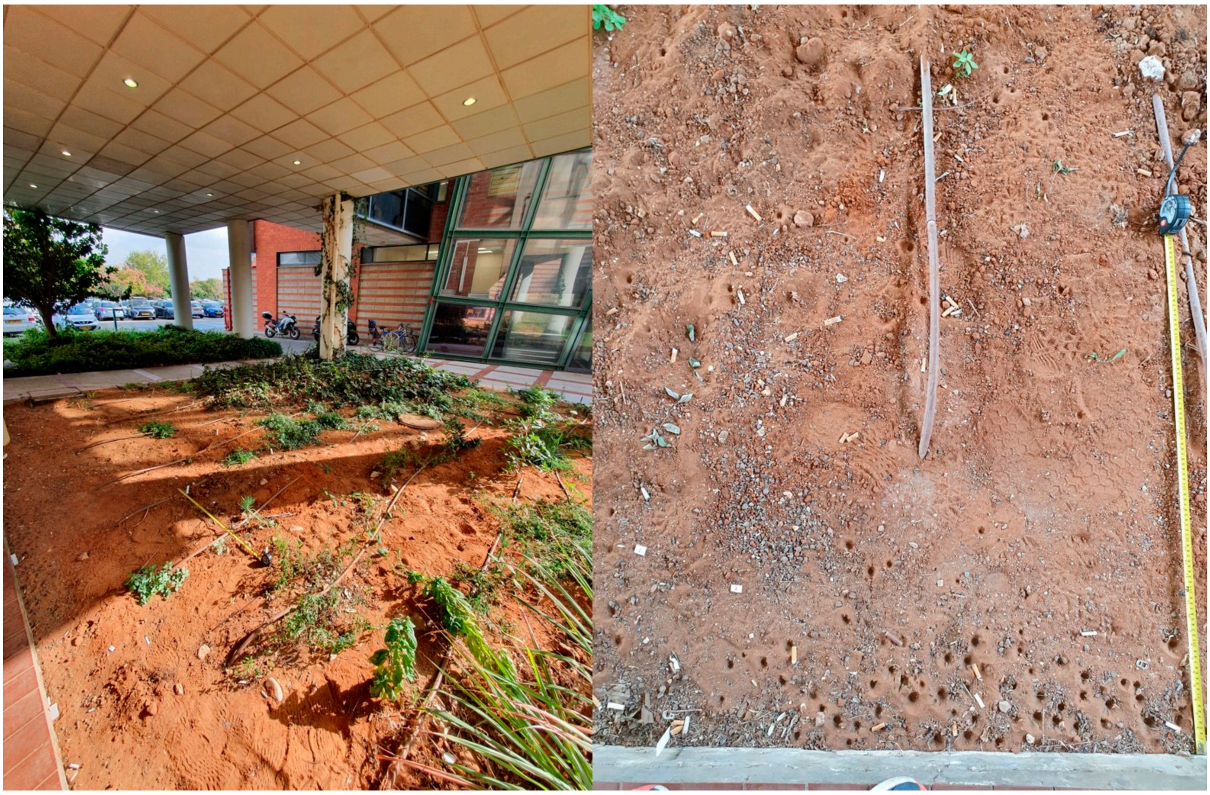

Figure S1f: Mexico.

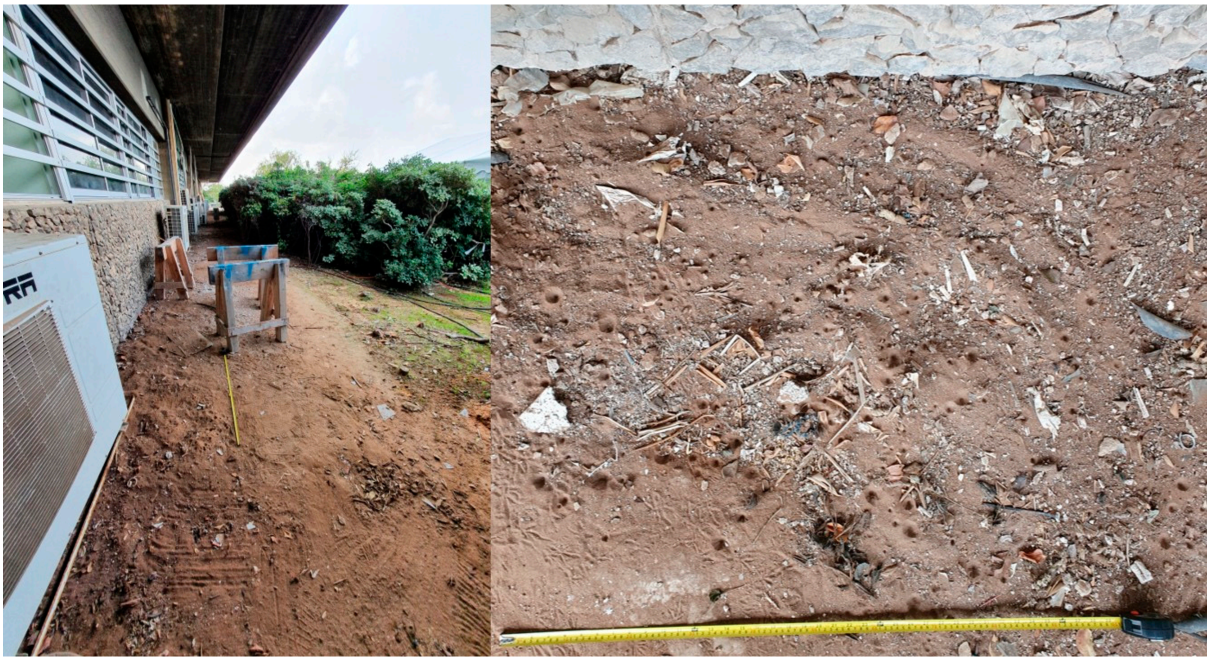

2. Wormlion pits constructed in the field vs. the lab:

It is important to compare animal behavior in the field and in the lab (~22ºC, natural day:night cycle) in order to verify that factors were discovered to have a significant effect in the lab also hold under natural settings. We compare here pit areas of wormlions constructed under these two settings, with an interval of a day in-between. We used four Pearson correlations, separately for each of the four sites examined. Then, we examined whether the r values differed among sites (a test to compare correlations from independent samples). Pit areas were square-root transformed due to their deviation from a normal distribution.

Britannia: $r = 0.502$, $N = 21$, $P = 0.020$.
Green: $r = 0.617$, $N = 24$, $P = 0.001$.
Mexico: $r = 0.779$, $N = 20$, $P < 0.001$.
Schreiber: $r = 0.499$, $N = 25$, $P = 0.011$.
Comparing the correlations of the two sites differing the most in their r values indicated there is no significant difference in their r values (Schreiber vs. Mexico: $z = -1.532$, $P = 0.063$).

Figure S2: Areas of wormlion pits constructed in the field and the lab in four sites at Tel Aviv University: (A) Britannia, (B) Green, (C) Mexico, and (D) Schreiber buildings.

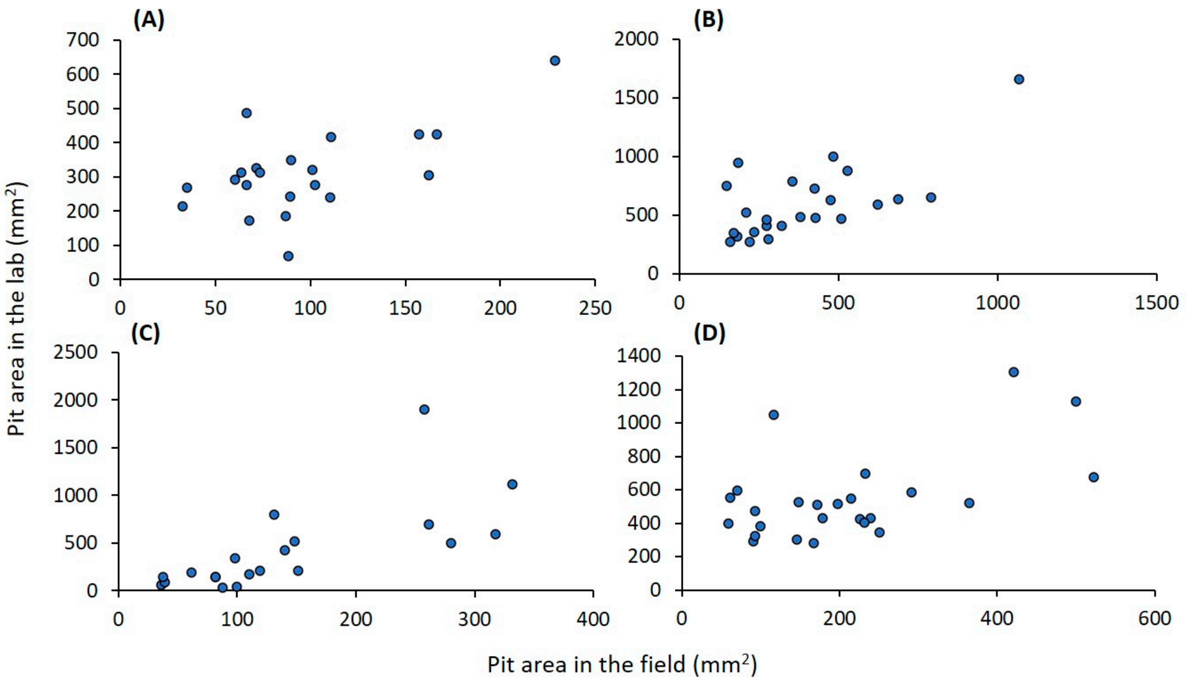

3. Pitfall traps to estimate the arthropod abundance:

Pitfall traps were placed in two sites, next to the Green and Schreiber buildings, at Tel Aviv University.

Figure S3: The pitfall traps, placed either next to the wall or in a distance of 40 cm.

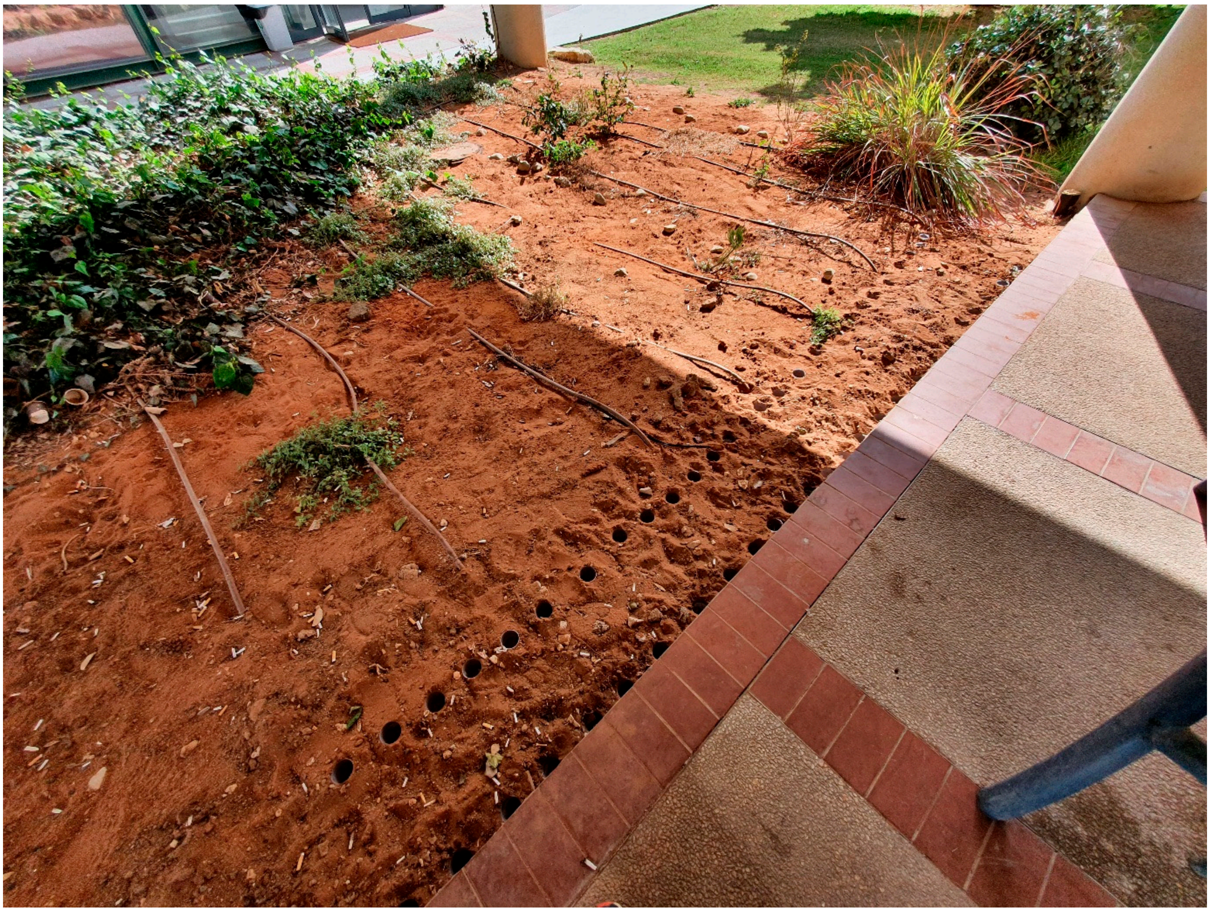

Figure S4: A flowchart of the simulation model.

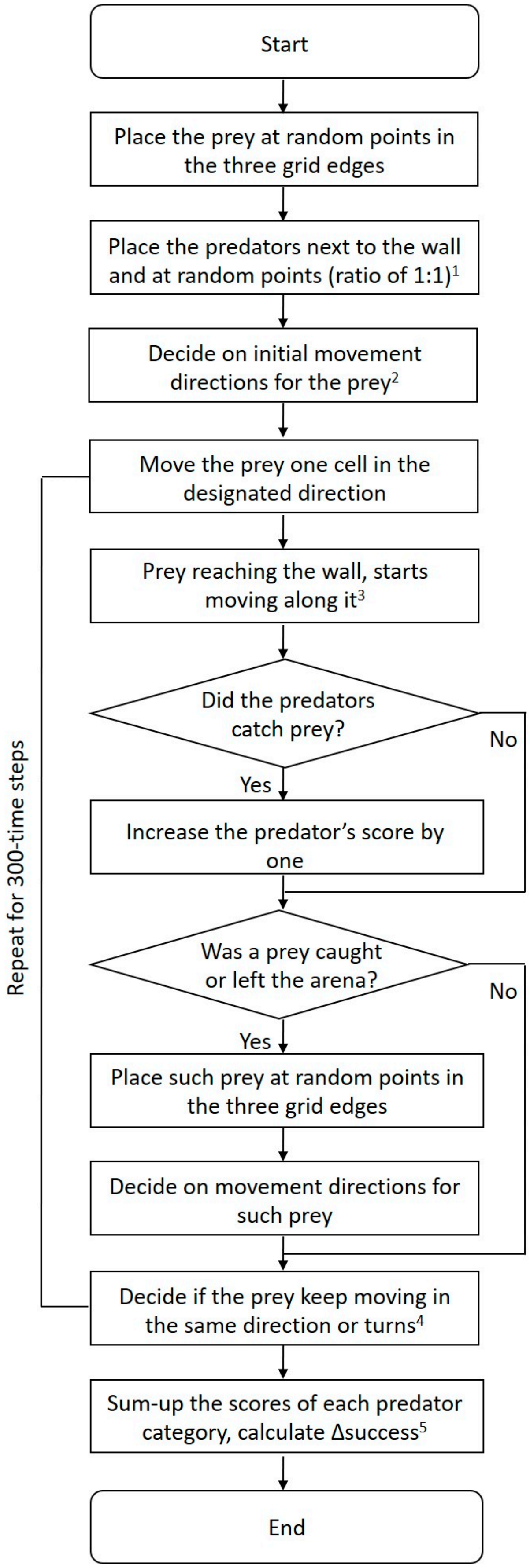

4. A flowchart of the simulation model.

[1] Overlaps among predator locations are not allowed.

[2] Movement is possible in one of four directions (forward, backward, left, right).

[3] In one of the two possible directions, randomly chosen.

[4] The prey can turn in 90º either right or left in respect to its current movement direction.

[5] Δsuccess = number of prey caught by the predators next to the wall – number of prey caught by other predators.

5. Results/Statistics for the link between the distance from the wall, body mass, soil depth, and pit area in the field and the lab.

Table S1:

Site	Distance → Mass (t, P value)	Distance, Mass & Depth → pit field (t, P value)	Distance, Mass & Depth → pit lab (t, P value)
Britannia	-2.548, **0.018**	Dist.: -0.103, 0.919<br>Depth: 0.704, 0.489<br>Mass: 1.817, 0.084<br>Mass*: 3.164, **0.004**	Dist.: -0.053, 0.958<br>Depth: 1.184, 0.253<br>Mass: 1.424, 0.173<br>Mass*: 2.777, **0.012**
Green	-1.207, 0.240	Dist.: 1.841, 0.080<br>Depth: 1.067, 0.298<br>Mass: 3.000, **0.007**	Dist.: -0.288, 0.777<br>Depth: 1.159, 0.260<br>Mass: 2.353, **0.029**
Mexico	0.203, 0.841	Dist.: 3.496, **0.002**<br>Depth: 0.648, 0.524<br>Mass: 7.179, <**0.001**	Dist.: 1.274, 0.221<br>Depth: 0.190, 0.852<br>Mass: 8.847, <**0.001**
Schreiber	-0.055, 0.956	Dist.: -1.197, 0.245<br>Depth: 0.762, 0.455<br>Mass: 2.220, **0.038**	Dist.: 0.937, 0.359<br>Depth: -0.246, 0.808<br>Mass: 2.036, 0.055<br>Mass*: 2.105, **0.046**

Mass*: When the non-significant variables are removed and the test is redone. N = 25 for the second and third columns. N = 21, 24, 20, and 25 for each site in the order of appearance. Significant results are in bold.

6. Results/Statistics for the change in illumination with daytime in late May (M=Morning, N=Noon, A=Afternoon) and the difference between positions next to the walls and distant from the walls (W, D). At each site, the three rows stand for each explanatory variable tested: Time (df = 2, 24), Position (1, 24), and their interaction (2, 24). The order of letters indicates how daytimes and positions differ (larger values first). This table is similar to Table 2 in the manuscript, but refers to the second measurement in late May, instead of the first measurement in early March. The results regarding the differences between positions next to the wall and at a distance of 40 cm across sites are qualitatively identical in March and May. There are nevertheless some differences among daytimes, which are expected due to the longer light hours in May than March.

Table S2:

**Site**	**F**	**P**
Britannia	306.315	**<0.001** N, A, M
	19.459	**<0.001** D, W
	0.841	0.444
Buchmann-Mehta	113.114	**<0.001** A, N, M
	18.055	**<0.001** W, D
	6.466	**0.006**
Central library	23.948	**<0.001** A, N, M
	<0.001	0.994
	0.468	0.632
Gilman	8.286	**0.002** A, M, N
	1.978	0.172
	0.233	0.794
Green	636.615	**<0.001** A, N, M
	293.898	**<0.001** D, W
	114.877	**<0.001**
Mexico	14.256	**<0.001** M, N, A
	5.452	**0.028** D, W
	0.245	0.785
Schreiber	989.562	**<0.001** N, M, A
	112.151	**<0.001** D, W
	43.921	**<0.001**

We chose four sites, in which the differences in illumination between positions next to the wall and positions 40 cm from the wall were the greatest: Britannia and Schreiber at noon, and Green and Buchmann-Mehta afternoon. In the latter site, positions next to the wall were more lit, whereas in all other sites, those next to the wall were more shaded. We measured temperature next to the wall in mid-June 40 cm from the wall, and 100 cm from the wall, using an alcohol thermometer, placed ~0.5 cm below the ground. These measurements suggest a link between illumination and ground temperature, as expected.

Table S2a:

**Site (Orientation), Time**	**Wall**	**40 cm**	**100 cm**
Britannia (S), noon	24.5-25.0°C (shaded)	24.5-25.0°C (shaded)	37.5-38.0°C (lit)
Schreiber (E), noon	25.0-25.5°C (shaded)	32.0-32.5°C (shaded)	42.0-42.5°C (lit)
Green (S), afternoon	25.0-25.5°C (shaded)	26°C (shaded)	27.0-27.5°C (shaded)
Buchmann-Mehta (W), afternoon	29.0-29.5°C (lit)	27.0-27.5°C (partial shade)	27.0-27.5°C (partial shade)
